# Factors associated with delay or avoidance of medical care during the COVID-19 pandemic in Armenia: results from a nationwide survey

**DOI:** 10.1186/s12913-023-10483-x

**Published:** 2024-01-04

**Authors:** Serine Sahakyan, Diana Muradyan, Aida Giloyan, Tsovinar Harutyunyan

**Affiliations:** Turpanjian College of Health Sciences, American University of Armenia, 40 Marshal Baghramian Ave, Yerevan, 0019 Armenia

**Keywords:** Healthcare systems, Critical care, Primary care, Accessibility of health services

## Abstract

**Background:**

The coronavirus disease 2019 (COVID-19) pandemic has disrupted healthcare systems throughout the world. Many patients faced delays and cancellation of care due to scaled back services, mobility restrictions, and concerns related to the risk of infection. The present study aimed to assess the prevalence of and risk factors associated with the avoidance or delay of medical care due to COVID-19 in Armenia.

**Methods:**

We conducted a cross-sectional telephone survey of 3,483 adults across Armenia. We used stratified two-stage cluster sampling to select the participants from different age groups proportionate to their size in the population. Logistic regression analysis assessed the association of risk factors with avoidance/delay of routine, urgent/emergency, and any medical care.

**Results:**

The mean age of the sample was 49.5 (SD = 14.8), ranging from 18 to 90. About 9.9% of the respondents avoided/delayed any type of medical care; whereas 5.5% avoided/delayed urgent/emergency care and 6.6% routine care. In the adjusted analysis, female gender and higher monthly expenditures were associated with avoidance/delay of routine medical care. Factors associated with delay/avoidance of urgent/emergency care included female gender and higher perceived threat of COVID-19. Younger age, female gender, higher perceived threat and not being vaccinated against COVID-19 were associated with avoidance/delay of any medical care in the adjusted analysis.

**Conclusion:**

Since avoiding or delaying care might increase morbidity and mortality associated with conditions not related to COVID-19, identifying population groups that are more likely to avoid care is important. Targeting such groups with educational interventions focusing on the risks of using versus not using medical care in times of pandemic might be crucial. Ensuring the provision of in-home healthcare services for high-risk groups might help to address important medical care needs during the pandemic.

## Background

The coronavirus disease 2019 (COVID-19) pandemic has disrupted healthcare systems throughout the world [1]. The unprecedented pressure on the health sector necessitated diverting attention and resources from non-COVID-19 care to the management of COVID-19 cases [2]. As healthcare providers scaled back some of the services, many patients encountered delays in the treatment of non-COVID-19-related conditions [3, 4]. Some patients avoided seeking care for several reasons including reduced trust in both the healthcare system’s handling of COVID-19 and non-COVID-19 issues, income loss during the pandemic, experiencing negative emotions due to social distancing measures, and having a fear of contracting COVID-19 as one of the most commonly reported reasons [5–7]. Also, lockdowns introduced in many countries to control the spread of the infection affected people’s mobility and limited their access to care [8].

A study conducted in the US in 2020 reported that an estimated 41% of the US population delayed or avoided medical care during the pandemic because of COVID-19-related concerns [6]. According to the same study, 12% of the respondents avoided emergency care, while 31.5% avoided routine care. The avoidance and delay of medical care can limit the opportunities to manage chronic conditions or detect new diseases early [6]. Moreover, avoidance of medical care could increase the risk of morbidity and mortality associated with illnesses that can be treated and prevented, and contribute to excess deaths [9]. For example, the reduction of breast cancer screening during the pandemic period in the US, Spain and Canada due to limited access to primary health-care as well as fear of contracting COVID-19, contributed to the decrease in new diagnoses of breast cancer [10–12]. The recent meta-analysis for head and neck, colorectal, breast, and lung cancers [13] demonstrated that even a one-month surgery and radiotherapy delay can result in a high likelihood of the risk of death (hazard ratio of 1.06–1.08; and 1.09 correspondingly).

Other studies have shown that the number of hospital admissions and emergency department visits has also substantially declined in the US early in the pandemic [14, 15]. Notably, deaths at home from acute coronary syndrome have risen due to avoidance of urgent medical care because of the fear of contracting COVID-19 in the hospital [9, 16].

The risk of avoidance of medical care appears to be distributed unevenly across the population. Studies conducted mostly in higher-income Western countries have shown that avoidance of any medical care because of concerns related to COVID-19 is more prevalent in younger adults [6, 17], females [6, 18, 19], people having comorbidities and disabilities [6, 19], unpaid caregivers [6], those with high level of perceived threat of COVID-19 [18, 19], and those with depression and anxiety [20].

The change in healthcare utilization patterns due to pandemic and the factors associated with forgoing medical care in other parts of the world have been less frequently explored. The study aimed to assess the prevalence of and risk factors associated with the avoidance or delay of medical care due to COVID-19 in Armenia.

### Situation in Armenia

In Armenia, primary health-care (PHC) services are provided by around 337 public and private health facilities, including polyclinics, rural ambulatories and health posts, and PHC units at medical centers [21]. Since 2006, the Basic Benefit Package (BBP) provides free PHC for the entire population but excludes costly diagnostic care and certain medications. It is common in Armenia to self-refer to hospitals and urgent care rather than seek PHC services due to perceptions about the low quality of PHC services [22]. The PHC service utilization remains low compared to European countries. In 2017, for example, the average number of PHC visits in Armenia was 4.1 [23], which is substantially lower than the average of 7.6 within the WHO European Region [24]. In contrast, the utilization of ambulance services in Armenia is one of the highest in the world. It receives approximately 600 calls per day, of which 25% are considered true emergencies [25].

The first case of COVID-19 in Armenia was reported on March 1 2020 [26]. Before mid-May 2020 all suspected cases were quarantined in dedicated health facilities until negative test results were confirmed. Those who had positive test result, depending on the symptoms, were quarantined in hotels or health facilities, and those patients without symptoms were isolated in dedicated facilities (repurposed hotels) [26]. The strategy of handling the cases was changed due to the rising number of COVID-19 daily cases between May 2020 and September 2021. PHC providers and the dedicated hotline served as the first point of contact. A 14-day self-isolation and monitoring was established for asymptomatic and mild COVID-19 confirmed cases. They were informed about the prevention of the spread of infection and the symptoms of COVID-19, and instructed to immediately contact the PHC physician in case of deterioration of their health condition [27]. All moderate cases (pneumonia without hypoxia) were treated by PHC physician and monitored at home until the regression of symptoms. All severe cases were hospitalized.

COVID-19 vaccination in Armenia became available in April of 2021. At the start of the data collection for the present study, in May 2021, Armenia had more than 192 thousand cases and about 3,500 deaths, while the proportion of vaccinated people (those who received at least one dose) was 1.1%. As of May 2022, around 2,150,000 doses of vaccine were administered [28].

A qualitative study conducted in June 2021 among the general population and health-care providers in Armenia has captured a perception that the provision of essential health services in the PHC system during the pandemic suffered significantly [29]. In particular, the restrictions implemented during the lockdown, as well as population’s fears related to the transmission of the infection at the health care facilities led to a decrease in outpatient visits among people with chronic conditions [29].

## Methods and materials

We conducted a cross-sectional telephone survey of 3,483 adults 18 years old or older in the capital city Yerevan, where more than 36% of the population resides [30], and 10 marzes (provinces). The survey was one of the components of a larger study with the primary aim of estimating SARS-CoV-2 seroprevalence in the Armenian population, which was completed in the scope of the USAID-funded “Support to Control COVID- 19 and Other Infectious Disease Outbreaks” project by the College of Health Sciences of the American University of Armenia (AUA/CHS) [31]. The methods have been described in full elsewhere [32]. Sample size calculation was performed using the formula for estimating population proportion [33] and was based on the assumption of 30% prevalence of antibodies against SARS-CoV-2, 1.45% margin error, 0.05 type I error, and 95% confidence level. The required sample size was estimated to be 3,832. The exclusion criteria for the study were having a contraindication to venipuncture, being in quarantine or self-isolation for less than 10 days, or being unable to attend the sampling site because of physical disability. Out of the total sample enrolled in the blood sampling stage, 3,483 adults participated in the phone survey.

The study participants were selected via PHC facilities using stratified two-stage cluster sampling. PHC facilities cover about 97% of the Armenian population [21]. The participants were recruited from the different age groups proportionate to their size in the general population.

Ten trained interviewers completed the phone survey using electronic tablets with the Alchemer online tool [34]. The full study protocol was pretested prior to data collection.

The main outcome variable of interest for this analysis was the delay or avoidance of medical care, which was assessed through the following questions with answer options “yes” and “no”: “Have you ever delayed or avoided urgent medical care due to concerns related to COVID-19 (for example, to avoid getting infected with COVID-19)”? and “Have you ever delayed or avoided routine/planned medical care (for example, regular check-up, vaccination, etc.) due to concerns related to COVID-19”? A combined variable was created with the following categories: avoidance/delay of routine medical care, avoidance/delay of urgent/emergency medical care, and avoidance/delay of any medical care (either routine, urgent or both routine and urgent).

Participants’ nationality (Armenians vs. others), place of residence (city vs. villages), age, gender (males vs. females), education (incomplete secondary (< 10 years), complete secondary/vocational (≥ 10-≤13 years), university/postgraduate (> 13 years), employment status (employed, unemployed and retired), monthly expenditure (less than 100,000 Armenian drams (AMD), (~ USD 250), from 101,000 to 200,000 AMD (~ USD 250–500), from 201,000 to 400,000 AMD (~ USD 500–1000), and above 401,000 AMD (~ USD 1000), and COVID-19 vaccination status (vaccinated vs. not vaccinated) were the independent socio-demographic variables.

In addition, we assessed the perceived threat of COVID-19 using the following questions: (1) How susceptible do you consider yourself to an infection with COVID-19 and (2) How severe would contracting COVID-19 be for you (how seriously ill do you think you would be)? The answer options ranged from zero (not at all susceptible) to three (very susceptible) for both variables. The responses to these two questions were summed up to calculate the perceived threat score ranging from 0 to 6. The higher score indicated a higher perceived threat. The presence of thirteen chronic conditions was assessed with a question with multiple response options. A summative score indicating the number of chronic conditions present in the respondents was generated for the analysis.

The Statistical Package for Social Sciences (SPSS) version 23.0 was used to analyse the data (SPSS inc., Chicago, IL, USA). We performed descriptive analysis using means and standard deviations for continuous variables and percentages for categorical variables. Simple and multivariable logistic regression explored the associations between independent variables and the outcome variables. We run three logistic regression models to assess the association of risk factors with avoidance/delay of routine, urgent/emergency, and any medical care. All variables associated with avoidance/delay of medical care at the p < 0.05 level in the bivariate analysis were entered into multivariable models.

We checked basic assumptions for logistic regression before running the analysis.

The American University of Armenia Institutional Review Board and WHO Ethical Research Committee (ERC)/ COVID-19 approved the study protocol (#AUA-2021-005; #WHO ERC-CERC.0112).

## Results

### Descriptive analysis

The mean age of the study participants was 49.5 (SD = 14.8), ranging from 18 to 90. Females constituted about 71% of the sample. Approximately 39% of the sample reported university or higher education, 31% reported vocational education, and 29% incomplete or secondary education (Table 1). The majority of the respondents have not received the COVID-19 vaccine (88.4%). The mean number of chronic conditions in the sample was 0.94 (SD = 1.09). About 10% (345) of the respondents avoided or delayed (avoidance/delay) any type of medical care; whereas 5.5% (192) avoided or delayed urgent and or emergency care (urgent/emergency) and 6.6% (231) routine care (Table 1).

**Table 1:** Socio-demographic and health characteristics, and perceived threat of COVID-19 by avoidance/delay of any type of medical care for COVID-19

Variables	Total, n = 3,483	Avoidance/delay of routine medical care, (n = 231)	Avoidance/delay of urgent medical care, (n = 192)	Avoidance/delay of any type of medical care, (n = 345)
	**n (%)**	n (%)	n (%)	n (%)
**Nationality**				
Armenians	3,442 (99.0)	229 (6.8)	191 (5.7)	342 (10.2)
Others^a^	36 (1.0)	2 (5.6)	1 (2.9)	3 (8.6)
**Place of residence**				
City	3,075 (88.5)	207 (6.9)	170 (5.7)	305 (10.2)
Village	400 (11.5)	22 (5.6)	22 (5.6)	38 (9.7)
**Age** (mean, (SD))	49.5 (14.8)	47.6 (14.3)	47.5 (13.9)	47.8 (14.2)
**Gender**				
Male	1,011 (29.0)	44 (4.5)	36 (3.7)	66 (6.8)
Female	2,472 (71.0)	187 (7.7)	156 (6.4)	279 (11.5)
**Education**				
Incomplete secondary	134 (3.9)	6 (4.7)	7 (5.5)	11 (8.7)
Complete secondary	888 (25.6)	53 (6.2)	47 (5.5)	82 (9.6)
Vocational	1,089 (31.4)	57 (5.4)	54 (5.1)	92 (8.7)
University	1,293 (37.3)	107 (8.4)	76 (6.0)	148 (11.7)
Postgraduate	60 (1.7)	8 (13.6)	8 (13.6)	12 (20.3)
**Employment**				
Employed^b^	1,882 (54.4)	117 (6.4)	110 (6.0)	184 (10.0)
Unemployed^c^	1,036 (30.0)	77 (7.6)	61 (6.0)	113 (11.1)
Retired	541 (15.6)	35 (6.7)	20 (3.8)	46 (8.8)
**Monthly expenditure (Armenian dram - AMD)**				
Less than 200,000	1834 (65.78)	106 (5.92)	112 (6.27)	179 (10.02)
From 201,000 to 300,000	640 (22.96)	49 (7.83)	44 (7.02)	71 (11.34)
Over 300,000	314 (11.26)	34 (11.07)	16 (5.18)	42 (13.68)
**Perceived threat (susceptibility and severity)**, *mean, SD*	3.12 (1.22)	3.3 (1.2)	3.3 (1.2)	3.3 (1.2)
**Number of chronic conditions**,^d^* mean (SD)*	0.94 (1.09)	1.0 (1.1)	1.0 (1.2)	1.1 (1.2)
**COVID-19 vaccination status**				
Vaccinated	393 (11.6)	18 (4.6)	15(3.8)	27 (6.9)
Not vaccinated	2,993 (88.4)	212 (7.1)	175 (5.9)	316 (10.6)

^a^ Russian, Yazidi, Lebanese, Ukrainian, Polish

^b^ Including work from home, own business, seasonal work in foreign countries, farmers, maternity leave

^c^ Unemployed includes also students and military officers

^d^ Obesity, cancer, diabetes mellitus, AIDS/ diseases of immune system, heart diseases, asthma, chronic lung diseases, chronic liver diseases, chronic blood diseases, chronic kidney diseases, chronic neurological diseases, organ/bone marrow transplantation and other (ophthalmological, rheumatic, urological etc.)

### Simple and multivariable logistic regression analysis

Simple logistic regression analysis showed that female gender was associated with avoidance/delay of routine care, urgent medical care, and any care. Higher monthly expenditure was associated with the avoidance/delay of routine medical care only. Perceived threat of COVID-19 was associated with the avoidance/delay of urgent/emergency medical care and any care. Age, having non-communicable diseases and COVID-19 vaccination status were associated with avoidance/delay of any medical care in the bivariate analysis (Table 2).

**Table 2:** The results of simple logistic regression analysis of factors associated with avoidance/delay of routine, urgent, and any type of medical care during COVID-19 in Armenia

	Outcome 1		Outcome 2		Outcome 3	
	Routine medical care		Urgent/emergency medical care		Any medical care ^a^	
Independent variables	OR (95% CI)	p-value	OR (95% CI)	p-value	OR (95% CI)	p-value
**Age (years)**	0.99 (098; 1.00)	0.054	0.99 (0.98; 1.00)	0.07	0.99 (0.98; 0.99)	**0.033**
**Gender**						
Male	1.00		1.00		1.00	
Female	1.77 (1.26; 2.48)	**0.001**	1.79 (1.24; 2.59)	**0.002**	1.79 (1.35; 2.37)	**< 0.001**
**Nationality**						
Others	1.00		1.00		1.00	
Armenians	1.25 (0.30; 5.22)	0.76	2.05 (0.28; 15.1)	0.48	1.21 (0.37; 3.98)	0.75
**Place of residence**						
Village	1.00		1.00		1.00	
City	1.25 (0.80; 1.97)	0.33	1.01 (0.64; 1.60)	0.96	1.06 (0.74; 1.51)	0.76
**Education**						
Incomplete secondary	1.00					
Complete secondary and vocational	1.22 (0.53; 2.84)	0.64	0.95 (0.43; 2.09)	0.90	1.05 (0.56; 1.99)	0.88
University and postgraduate	1.91 (0.82; 4.44)	0.13	1.16 (0.52; 2.56)	0.72	1.45 (0.76; 2.74)	0.26
**Employment**						
Retired	1.00					
Employed	0.95 (0.64; 1.40)	0.78	1.60 (0.98; 2.60)	0.06	1.15 (0.82; 1.62)	0.41
Unemployed	1.14 (0.75; 1.73)	0.53	1.60 (0.96; 2.68)	0.07	1.29 (0.90; 1.86)	0.16
**Monthly expenditure** * (Armenian dram - AMD)*						
Less than 200,000	1.00		1.00		1.00	
From 201,000 to 300,000	1.35 (0.95; 1.92)	0.10	1.13 (0.79; 1.62)	0.51	1.15 (0.86; 1.54)	0.35
Over 300,000	1.98 (1.32; 2.97)	**0.001**	0.82 (0.48; 1.40)	0.46	1.42 (0.99; 2.04)	0.06
**Perceived threat of COVID-19**	1.09 (0.96; 1.23)	0.17	1.16 (1.02; 1.32)	**0.025**	1.11 (1.01; 1.23)	**0.040**
**Number of chronic non-communicable diseases** * (ranging from 1 to 13)*	1.09 (0.97; 1.22)	0.16	1.06 (0.93; 1.20)	0.43	1.12 (1.02; 1.23)	**0.023**
**Covid-19 Vaccination status**						
Not vaccinated	1.00		1.00		1.0	
Vaccinated	0.63 (0.38; 1.03)	0.06	0.64 (0.37; 1.10)	0.10	0.63 (0.42; 0.94)	**0.024**

^a^Delayed/ avoided either routine, urgent or both routine and urgent medical care

Statistically significant p-values are indicated in bold

In the adjusted analysis, the associations of female gender (OR = 1.80, 95% CI: 1.24–2.63, p = 0.002) and monthly expenditure over 301,000 AMD (OR = 2.13, 95% CI: 1.41–3.21, p < 0.001) with avoidance/delay of routine medical care were maintained (Model 1). Female gender (OR = 1.78, 95% CI: 1.19–2.67, p = 0.005) and perceived threat (OR = 1.15, 95% CI: 1.01–1.31, p = 0.036) were associated with avoidance/delay of urgent/emergency medical care (Model 2). Age (OR = 0.99, 95% CI: 0.98-1.00, p = 0.049), female gender (OR = 1.77; 95% CI: 1.29–2.44, p < 0.001), perceived threat (OR = 1.12, 95% CI: 1.00-1.24) and having been vaccinated against COVID-19 (OR = 0.53; 95% CI: 0.33–0.86, p = 0.010) were associated with avoidance/delay of any type of medical care in the adjusted analysis (Model 3), (Table 3).

**Table 3:** The results of multivariable logistic regression analysis of factors associated with avoidance/delay of routine, urgent, and any type of medical care during COVID-19 in Armenia

	Model 1		Model 2		Model 3	
	Routine medical care		Urgent/emergency medical care		Any medical care ^a^	
Independent variables	OR (95% CI)	p-value	OR (95% CI)	p-value	OR (95% CI)	p-value
**Age (years)**					0.99 (0.98; 1.00)	**0.049**
**Gender**						
Male	1.00		1.0		1.0	
Female	1.80 (1.24; 2.63)	**0.002**	1.78 (1.19; 2.67)	**0.005**	1.77 (1.29; 2.44)	**< 0.001**
**Monthly expenditure (Armenian dram - AMD)**						
Less than 200,000	1.00					
From 201,000 to 300,000	1.40 (0.99; 2.00)	0.06				
Over 300,000	2.13 (1.41; 3.21)	**< 0.001**				
**Perceived threat of COVID-19**			1.15 (1.01; 1.31)	**0.036**	1.12 (1.00; 1.24)	**0.044**
**Number of chronic non-communicable diseases** * (ranging from 1 to 13)*					1.12 (0.99; 1.26)	0.07
**Covid-19 Vaccination status**						
Not vaccinated					1.0	
Vaccinated					0.53 (0.33; 0.86)	**0.010**

^a^Delayed/ avoided either routine, urgent or both routine and urgent medical care

Statistically significant p-values are indicated in bold

## Discussion

This study found that 5.5% and 6.6% of the study population avoided or delayed urgent/emergency and routine medical care during COVID-19 pandemic in Armenia, respectively. The rates of avoidance or delay of both urgent/emergency and routine care in our study are lower compared to the findings from a survey conducted among the US adult population, which reported 41% delayed or avoided of medical care, including 12% urgent and 32% routine care [6], and the findings from the study, conducted among Australian adult population, which reported that 26.4% of the study population avoided/delayed routine medical care and 10.1% urgent/ emergency care [19]. A longitudinal study conducted in the Netherlands found that 7.0% and 6.4% of Dutch people cancelled primary care appointments and hospital outpatient appointments during COVID-19 pandemic, respectively [35]. The lower rate of avoidance or delay found in our study could be explained, at least partially, by the low healthcare utilization rates in Armenia which have been documented even prior to COVID-19 pandemic [36, 37]. At the same time, this finding might suggests that Armenians have not been heavily restricted in their use of health-care services specifically due to COVID-19 compared to the residents of other more developed countries.

In the present study women had significantly higher odds of avoidance/delay of either routine or urgent/emergency medical care compared to men, which is consistent with the literature. A study conducted among adult population aged over 18 years in Australia, reported that the avoidance of any medical care was significantly higher among women compared to men during the COVID-19 pandemic (adjusted prevalence ratio (aPR) = 1.30, 95% CI = 1.09–1.55) [6]. The similar finding was reported in the study, conducted among residents of the Ommoord district in Rotterdam aged 55 years and older [18]. One of the possible reasons of delaying medical care due to COVID-19 concerns by women could be increased caretaking responsibilities during the pandemic and concerns about exposing their household members to the virus if they catch it at the healthcare facility. As a result, women have experienced worsening health conditions due to skipping healthcare services during the pandemic [38]. In fact, the link between avoidance of both emergency and routine medical care during the pandemic and caregiving has been reported by other authors [6]. We, however, have not specifically explored caregiving status in the present study. Another explanation for delayed medical care in women could be the higher rates of healthcare utilization among women as compared to men in general [39]. Since women are more likely to seek care they might have to schedule and delay medical care more often than men [17, 40].

We found that with each one-year increase in age, the adjusted odds of avoidance/delay of medical care significantly decreased in our sample. This finding is in line with findings from previous studies exploring care utilization patterns during COVID-19. For example, in a study conducted among US older population to assess socio-demographic factors associated with the delay of any medical care during COVID-19, people aged over 75 were less likely to avoid medical care compared to those aged 55 to 74 (OR 0.57; 95% CI 0.45–0.71) [17], while Gertz et al. reported that people aged over 65 had lower odds of delaying medical care compared to those aged between 45 and 64 during COVID-19 (OR = 0.88, 95% CI (0.80–0.96) [41]. One of the possible explanations of this phenomenon could be a higher share of difficult-to-delay mandatory/critical appointments among older people as compared to that in the younger population.

COVID-19 vaccination status was significantly associated with avoidance/delay of any type of care, with unvaccinated people more likely to avoid/delay medical care than those who were vaccinated. Since the perceived threat of COVID-19 was controlled in our adjusted analysis, this result is difficult to interpret. Those who have not been vaccinated likely had certain characteristics that were not part of our analysis that made them more inclined to avoid/delay care. While the literature on possible factors explaining this association is scarce, some authors assume that the overall attitude towards preventive and routine care could explain both the reluctance or inability to get vaccinated and the utilization of other types of care [42]. Also, geographic access, neighborhoods, characteristics of providers and social relations have all been shown to affect health service utilization patterns, yet none of them were explored in our study [42].

In our study, perceived threat was shown to influence the avoidance or delay of urgent/emergency care and any medical care. Several studies, conducted to assess the psychological factors associated with health-seeking behavior during the COVID-19 pandemic, support this finding [18]. Interestingly, in our study the perceived threat of COVID-19 was associated with avoidance of urgent care and not with the routine care. It might mean that those with higher perceived risk and severity of infection avoided or delayed care in hospital settings specifically. Studies have shown a sizeable risk of nosocomial transmission of COVID-19 in enclosed close-contact environments, such as hospitals [43]. The risk can be even higher in low-resource settings [43], and it might have been adequately grasped by the population. Globally, the number of heart attack patients seeking urgent hospital care has dropped by more than half during the pandemic, according to an extensive worldwide survey by the European Society of Cardiology [44]. Another study in the US found a significant drop in the number of cardiac patients during the pandemic [45].

In our study, people with an average monthly income of over 300,000 AMD (~ USD 600) had higher adjusted odds of delaying or avoiding routine medical care due to COVID-19 concerns compared to people with an average income of less than 200,000 AMD (~ USD 400). There are controversial findings on this association in literature. The possible explanation of our finding could be the preexisting low utilization level of primary health-care services by people with a high monthly income in our study population. Unfortunately, to our knowledge, no evidence has been collected in Armenia to date to support this hypothesis. However, studies in other countries have shown similar patterns. For example, in Germany, socially disadvantaged people visit general practitioners more often than more affluent individuals, who are more likely to visit a medical specialist [46]. This has been explained, at least partially, by a greater need for the services among poorer segments of society, but also by physician-patient interaction and the structural aspects of the health-care system [46]. In Armenia, PHC facilities are mainly providing routine health care for the general population free of charge. Poor quality perceptions have been identified as one of the barriers to primary care utilization in Armenia in previous studies [47]. It is also possible that people with higher socio-economic status were more knowledgeable about COVID-19 risks and protective factors and were more likely to avoid crowded settings as a result. The explanations on how socio-cultural and individual-level contexts interact with other factors and influence decision making about medical care utilization during COVID-19 are limited. Contextualized models of how people make decisions about seeking medical care during infectious disease outbreaks could further aid our understanding of the complex interplay of sociocultural and individual-level factors that could have influenced medical care utilization patterns during the COVID-19 pandemic [48].

The findings of this study are subject to several limitations. First, we relied on self-reported data which are prone to recall bias. Second, we did not specify the exact services implied under the “routine” or “emergency care” categories in the questionnaire, providing several examples of services in each category instead. We did not explore the need for services with a separate question; hence our respondents included both those who have not scheduled the services (hence have not canceled them) and those who wanted to use the services but had to avoid or delay them because of COVID-19. Finally, since the recruitment of participants was conducted in the scope of a larger seroprevalence study, we might have ended up with those respondents who were interested in knowing their seroprevalence status and were particularly concerned about COVID-19.

Future quantitative and qualitative studies should be conducted to assess the underlying factors of the association between average monthly income and the delay of routine medical care during pandemics. Behavioral characteristics of people with high income should be explored in terms of avoidance/delay of all types of medical care. Qualitative investigations exploring underlying reasons of medical care avoidance could provide in-depth information that could be used by policymakers to manage and control the utilization of health services during pandemics.

## Conclusion

This study supplies important information about care-seeking behavior during the pandemic. Since avoiding or delaying care might increase morbidity and mortality associated with conditions not related to COVID-19, identifying population groups that are more likely to avoid care and thus might be in need of targeted interventions, is important. Educating the public about the risks of using versus not using medical care in times of pandemic for different population groups might be crucial. Facilitation of the provision of in-home health-care services for those in high-risk groups might help to address important medical care needs during the pandemic.

## Data Availability

The datasets used and/or analyzed during the current study are available from the corresponding author on reasonable request.
